# Data on soil P^H^ of Barddhaman district, India

**DOI:** 10.1016/j.dib.2017.03.046

**Published:** 2017-04-08

**Authors:** Sumanta Bid

**Affiliations:** Dr. Bhupendra Nath Dutta Smriti Mahavidyalaya, Hatgobindapur, Burdwan 713407, Barddhaman, West Bengal, India

**Keywords:** Soil P^H^, Acidic soil, Alkaline soil, Fertility, Productivity

## Abstract

P^H^ (Puissance de Hydrogen) is an essential ingredient of soil that effects on fertility and productivity of dirt. Barddhaman district is a part of Lower Gangetic Plain fully covered by alluvial soil and popularly known as ‘rice bowl of West Bengal’ owing to its lofty production. This data article provides a block level data on soil P^H^ that is essential for further investigation of the relationship among soil ph, plant growth, plant health and productivity. This data is valuable in the field of soil geography and soil science. Soil P^H^ data is more relevant in the ground of plant biology, agricultural geography and agricultural science. It helps to explain the acidic and alkaline nature of alluvial soil. The data consist of 195 samples (*n*=195) taken from the entire district. Samples have been collected from March, 2014 to March, 2015 and experimented in the laboratory. Theoretically P^H^ value is limited within 0–14. Experiment result exemplifies the highest value 8.5 found in Khandaghosh block whereas lowest value is 4.5 and the samples which result in lowest value are gathered from 4 different blocks like Manteswar, Burdwan - II, Barabani and Salanpur.

**Specifications Table**

**Table:** 

Subject area	*Geography*
More specific subject area	*Soil Geography (pedology and edaphology), agricultural geography and soil science.*
Type of data	*Table, figure and excel data*
How data was acquired	*Through field work and laboratory experiment using soil P*^*H*^*measurement kit.*
Data format	*Raw*
Experimental factors	*Soil samples were collected from the depth of 25 cm throughout the complete year basically in three different periods – pre-monsoon, monsoon and post-monsoon.*
Experimental features	*Collected samples are rough in nature. They are processed individually through drying, pasting and separating. Dust and fine particles have extracted for laboratory experiment. Experiment is made manually using soil P*^*H*^*testing kit.*
Data source location	*195 coordinates recorded by GPS (Geographical Positioning System) of Barddhaman district, West Bengal, India.*
Data accessibility	*Data is available with this article.*

**Value of the data**

• The data provides a vivid picture about acidic and alkaline nature of alluvial dominated soil.
• It helps to explain the impact of soil P^H^ on the plant growth, soil fertility and productivity.
• Data can be utilized for quantitative analysis in the field of soil science and agricultural science.
• Other researchers may use the data for their research work and further analysis.

## 1 Data

Barddhaman district is broadly divided into two parts – north western part is known as industrial belt and eastern part is recognized as agricultural belt. The data presented here describes the soil P^H^ level of 39 different administrative units of the entire district. Data is given both in table and figure form and an excel data is also added in Supplementary material of the data article. The data is prepared on the basis of field work and laboratory experiment.

## 2 Experimental design, materials and methods

### 2.1 Sampling design, site selection and methods of sample collection

Barddhaman district is covered by 39 administrative units divided into 31 blocks, 6 municipalities and 2 municipal corporations. 5 samples are taken from each administrative area and the total number of sample *n*=195 (39×5=195). Sampling is designed on three different periods over the year – (a) Pre-monsoon period (March to mid June), (b) monsoon period (mid June to September) and (c) post-monsoon period (October to February) to observe the seasonal variation of P^H^ level. Two samples are taken in the pre-monsoon period, one sample in monsoon period and another two samples in post-monsoon period from each unit. A good sample is very much important for beneficial result. Samples are randomly collected giving emphasis on agricultural land and forest area. To ignore the exposed layer samples are gathered in depth of 25 cm from the soil surface which conserve the natural properties of it [1].

**Fig. 1:**
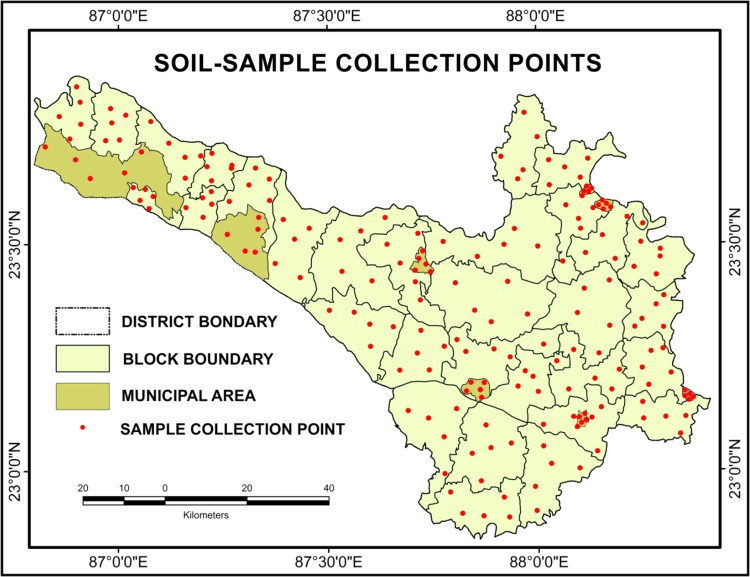
Sample collection points taken by handheld GPS.

Clean plastic containers are used to preserve the collected soil. Location coordinates of the sampling sites are acquired by the handheld GPS and projected on the georeferenced map made by the Arc GIS 9.3 version software (the permission for using the software is granted by the authority of The University of Burdwan) (Fig. 1). Collected raw materials are dried up for 7 days on the air temperature. Fine and dusty particles are prepared for laboratory experiment through pasting and filtering process respectively.

### 2.2 Laboratory experiment

**Fig. 2:**
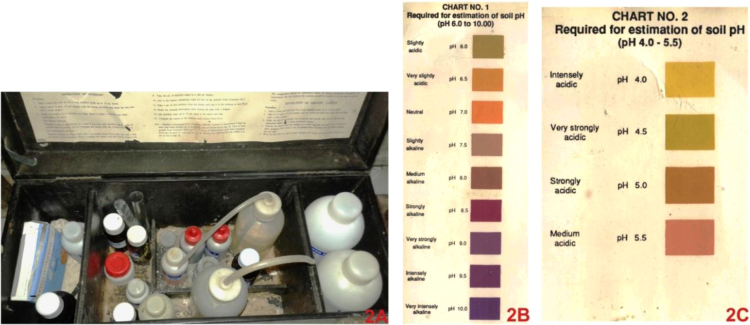
A. Soil P^H^ measuring kit; B. Chart 1 – P^H^ level 6.0 to 10.0; C. Chart 2 – P^H^ level 4.0–5.5.

Experiment procedure of P^H^ test using P^H^ testing kit (Fig. 2A) is [2] -

a. Take a clean test tube and pour distilled water up to 5 ml. mark.
b. Put 2 g of soil to the test tube.
c. Add 0.5 g of barium sulphate.
d. Allow the test tube to stand for 20 min with occasional shaking.
e. Add 5 drops of indicator no. 1 from container no. 1 to the above, close the mouth of the tube with a clean rubber stopper and shake the contents thoroughly. Allow the soil to settle down completely.
f. Compare the colour of the upper liquid in the test tube with the colour chart no. 1 (Fig. 2B) and find out the nearest match which will indicate its P^H^.
g. If the colour of the upper liquid in the test tube indicates P^H^ near 6 then repeat the whole experiment using indicator no. 2 instead of indicator no. 1 and match the colour of the upper liquid with the chart no. 2 (Fig. 2C).

### 2.3 Data registration

**Fig. 3:**
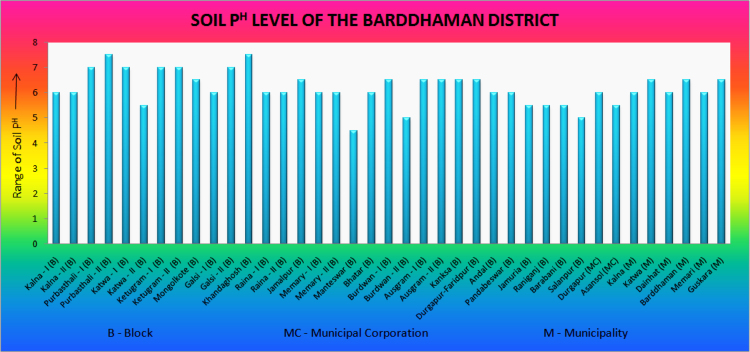
P^H^ data of different administrative units.

Experiment results are entered in sheet 1 of an excel file individually. Result of 5 samples for each administrative unit is converted into an average value. Average value is rounded in the nearest round figure of 0.5 intervals. Table and figure form of data are presented in the article (Table 1 and Fig. 3) and excel data is also available with it (Supplementary file).

Soil P^H^ is a crucial soil indicator and is defined as the negative log of the hydrogen ion activity [3]. The determination of soil P^H^ in soil is important as it plays a great role in availability of nutrients to plants [4]. Different P^H^ range indicates different types of acidic and alkaline soil [5] **(**Table 2**).**

## Figures and Tables

**Table 1:** 195 sampling data and average P^H^ level of 39 administrative units.

**Sl. No**	**Name of the Block/Municipality/Municipal Corporation**	**No. of Sample**	**Coordinate of the Sample Collection Point**	**P**^**H**^**Value**	**Average P**^**H**^**Value (Nearest Round Figure of 0.5 Interval)**
1	Kalna - I Block	1	23°13׳34׳׳N, 88°14׳58׳׳E	6.5	6.0
		2	23°11׳11׳׳N, 88°15׳53׳׳E	6.0	
		3	23°15׳42׳׳N, 88°16׳18׳׳E	6.0	
		4	23°12׳58׳׳N, 88°19׳03׳׳E	5.5	
		5	23°16׳59׳׳N, 88°17׳29׳׳E	6.0	
2	Kalna - II Block	6	23°07׳09׳׳N, 88°17׳29׳׳E	6.5	6.0
		7	23°09׳13׳׳N, 88°14׳53׳׳E	6.5	
		8	23°06׳01׳׳N, 88°15׳19׳׳E	5.5	
		9	23°07׳50׳׳N, 88°21׳20׳׳E	6.0	
		10	23°04׳49׳׳N, 88°20׳44׳׳E	5.0	
3	Purbasthali - I Block	11	23°23׳00׳׳N, 88°18׳39׳׳E	6.5	7.0
		12	23°21׳42׳׳N, 88°16׳35׳׳E	7.5	
		13	23°18׳51׳׳N, 88°18׳04׳׳E	6.5	
		14	23°18׳18׳׳N, 88°13׳53׳׳E	7.0	
		15	23°19׳25׳׳N, 88°14׳09׳׳E	7.0	
4	Purbasthali - II Block	16	23°25׳15׳׳N, 88°17׳20׳׳E	8.0	7.5
		17	23°26׳42׳׳N, 88°13׳54׳׳E	7.0	
		18	23°30׳11׳׳N, 88°14׳40׳׳E	7.5	
		19	23°29׳38׳׳N, 88°17׳49׳׳E	7.0	
		20	23°28׳01׳׳N, 88°17׳03׳׳E	8.0	
5	Katwa - I Block	21	23°35׳45׳׳N, 88°04׳32׳׳E	7.5	7.0
		22	23°34׳12׳׳N, 88°08׳11׳׳E	7.5	
		23	23°33׳28׳׳N, 88°05׳31׳׳E	6.5	
		24	23°27׳30׳׳N, 88°03׳40׳׳E	7.0	
		25	23°31׳35׳׳N, 88°06׳09׳׳E	7.0	
6	Katwa - II Block	26	23°33׳55׳׳N, 88°12׳23׳׳E	5.5	5.5
		27	23°28׳16׳׳N, 88°06׳15׳׳E	5.5	
		28	23°32׳51׳׳N, 88°15׳00׳׳E	5.5	
		29	23°27׳58׳׳N, 88°11׳41׳׳E	5.5	
		30	23°31׳11׳׳N, 88°09׳43׳׳E	6.0	
7	Ketugram - I Block	31	23°47׳38׳׳N, 87°58׳19׳׳E	7.5	7.0
		32	23°39׳26׳׳N, 87°58׳36׳׳E	7.5	
		33	23°44׳14׳׳N, 88°00׳15׳׳E	6.5	
		34	23°38׳14׳׳N, 87°57׳37׳׳E	7.0	
		35	23°41׳34׳׳N, 87°54׳51׳׳E	6.0	
8	Ketugram - II Block	36	23°41׳34׳׳N, 88°01׳39׳׳E	6.5	7.0
		37	23°41׳51׳׳N, 88°07׳37׳׳E	6.5	
		38	23°37׳52׳׳N, 88°01׳50׳׳E	6.5	
		39	23°40׳04׳׳N, 88°04׳55׳׳E	8.0	
		40	23°40׳03׳׳N, 88°04׳52׳׳E	7.0	
9	Mongolkote Block	41	23°31׳57׳׳N, 87°56׳44׳׳E	7.0	6.5
		42	23°28׳58׳׳N, 87°51׳32׳׳E	6.5	
		43	23°29׳13׳׳N, 88°00׳44׳׳E	6.5	
		44	23°30׳30׳׳N, 87°46׳40׳׳E	6.5	
		45	23°29׳11׳׳N, 87°55׳28׳׳E	6.5	
10	Galsi - I Block	46	23°19׳55׳׳N, 87°39׳20׳׳E	6.0	6.0
		47	23°21׳48׳׳N, 87°33׳20׳׳E	5.5	
		48	23°16׳54׳׳N, 87°36׳53׳׳E	6.0	
		49	23°21׳05׳׳N, 87°30׳14׳׳E	6.0	
		50	23°19׳34׳׳N, 87°35׳14׳׳E	6.5	
11	Galsi - II Block	51	23°18׳20׳׳N, 87°43׳12׳׳E	7.0	7.0
		52	23°13׳06׳׳N, 87°40׳17׳׳E	7.0	
		53	23°16׳07׳׳N, 87°46׳33׳׳E	7.0	
		54	23°13׳29׳׳N, 87°44׳28׳׳E	7.0	
		55	23°15׳58׳׳N, 87°42׳08׳׳E	7.5	
12	Khandaghosh Block	56	23°08׳57׳׳N, 87°41׳34׳׳E	8.5	7.5
		57	23°04׳40׳׳N, 87°46׳55׳׳E	8.0	
		58	23°08׳23׳׳N, 87°48׳26׳׳E	7.0	
		59	22°59׳41׳׳N, 87°46׳19׳׳E	7.0	
		60	23°07׳33׳׳N, 87°44׳11׳׳E	7.5	
13	Raina - I Block	61	23°06׳30׳׳N, 87°52׳49׳׳E	6.0	6.0
		62	23°02׳55׳׳N, 87°50׳25׳׳E	7.0	
		63	23°03׳31׳׳N, 87°56׳24׳׳E	6.0	
		64	22°58׳59׳׳N, 87°51׳53׳׳E	5.5	
		65	23°03׳09׳׳N, 87°53׳22׳׳E	5.5	
14	Raina - II Block	66	22°57׳01׳׳N, 87°47׳53׳׳E	5.5	6.0
		67	22°56׳44׳׳N, 87°55׳34׳׳E	6.5	
		68	22°54׳40׳׳N, 87°49׳11׳׳E	7.0	
		69	22°53׳45׳׳N, 87°55׳18׳׳E	5.5	
		70	22°54׳45׳׳N, 87°52׳14׳׳E	5.5	
15	Jamalpur Block	71	23°03׳46׳׳N, 88°00׳19׳׳E	6.5	6.5
		72	22°57׳39׳׳N, 87°59׳24׳׳E	7.0	
		73	23°01׳42׳׳N, 88°05׳45׳׳E	6.5	
		74	22°54׳48׳׳N, 87°59׳33׳׳E	6.0	
		75	23°00׳29׳׳N, 88°01׳31׳׳E	6.0	
16	Memary - I Block	76	23°06׳49׳׳N, 88°00׳54׳׳E	6.0	6.0
		77	23°06׳09׳׳N, 88°07׳02׳׳E	6.0	
		78	23°07׳30׳׳N, 88°05׳40׳׳E	5.5	
		79	23°02׳44׳׳N, 88°08׳23׳׳E	5.5	
		80	23°08׳13׳׳N, 87°41׳32׳׳E	7.0	
17	Memary - II Block	81	23°15׳51׳׳N, 88°05׳23׳׳E	6.0	6.0
		82	23°13׳53׳׳N, 88°11׳02׳׳E	7.0	
		83	23°10׳32׳׳N, 88°04׳15׳׳E	6.5	
		84	23°10׳31׳׳N, 88°10׳56׳׳E	6.0	
		85	23°12׳18׳׳N, 88°08׳02׳׳E	5.5	
18	Manteswar Block	86	23°15׳36׳׳N, 88°08׳48׳׳E	4.5	4.5
		87	23°24׳03׳׳N, 88°06׳00׳׳E	4.5	
		88	23°19׳07׳׳N, 88°10׳35׳׳E	5.0	
		89	23°25׳01׳׳N, 88°10׳47׳׳E	5.0	
		90	23°20׳14׳׳N, 88°05׳18׳׳E	4.5	
19	Bhatar Block	91	23°24׳45׳׳N, 87°48׳04׳׳E	6.0	6.0
		92	23°20׳59׳׳N, 87°58׳56׳׳E	5.5	
		93	23°21׳24׳׳N, 87°50׳04׳׳E	5.0	
		94	23°19׳00׳׳N, 87°53׳59׳׳E	6.5	
		95	23°25׳10׳׳N, 87°55׳17׳׳E	7.0	
20	Burdwan - I Block	96	23°17׳43׳׳N, 87°48׳20׳׳E	7.0	6.5
		97	23°15׳25׳׳N, 87°56׳15׳׳E	6.5	
		98	23°15׳13׳׳N, 87°49׳10׳׳E	7.0	
		99	23°16׳57׳׳N, 88°00׳12׳׳E	6.0	
		100	23°16׳00׳׳N, 87°53׳51׳׳E	6.0	
21	Burdwan - II Block	101	23°13׳52׳׳N, 87°58׳37׳׳E	5.0	5.0
		102	23°11׳41׳׳N, 87°57׳01׳׳E	5.0	
		103	23°14׳25׳׳N, 88°02׳26׳׳E	4.5	
		104	23°10׳58׳׳N, 88°00׳12׳׳E	4.5	
		105	23°12׳41׳׳N, 87°59׳17׳׳E	5.0	
22	Ausgram - I Block	106	23°31׳21׳׳N, 87°42׳42׳׳E	6.5	6.0
		107	23°25׳23׳׳N, 87°42׳18׳׳E	6.0	
		108	23°29׳20׳׳N, 87°38׳04׳׳E	6.0	
		109	23°22׳08׳׳N, 87°43׳29׳׳E	7.0	
		110	23°27׳07׳׳N, 87°40׳28׳׳E	7.0	
23	Ausgram - II Block	111	23°33׳26׳׳N, 87°38׳42׳׳E	6.0	6.5
		112	23°26׳29׳׳N, 87°32׳30׳׳E	7.5	
		113	23°31׳32׳׳N, 87°34׳49׳׳E	7.0	
		114	23°25׳08׳׳N, 87°36׳39׳׳E	6.0	
		115	23°30׳22׳׳N, 87°31׳10׳׳E	6.5	
24	Kanksa Block	116	23°33׳30׳׳N, 87°23׳18׳׳E	6.5	6.5
		117	23°27׳57׳׳N, 87°22׳44׳׳E	6.5	
		118	23°32׳35׳׳N, 87°27׳24׳׳E	7.0	
		119	23°25׳59׳׳N, 87°26׳26׳׳E	7.0	
		120	23°30׳20׳׳N, 87°25׳58׳׳E	6.5	
25	Durgapur–Faridpur Block	121	23°40׳53׳׳N, 87°19׳56׳׳E	6.0	6.5
		122	23°37׳26׳׳N, 87°18׳00׳׳E	6.5	
		123	23°38׳08׳׳N, 87°21׳35׳׳E	7.0	
		124	23°35׳18׳׳N, 87°15׳27׳׳E	6.5	
		125	23°35׳25׳׳N, 87°21׳07׳׳E	6.0	
26	Andal Block	126	23°37׳01׳׳N, 87°13׳54׳׳E	7.0	6.0
		127	23°35׳53׳׳N, 87°13׳24׳׳E	6.5	
		128	23°36׳00׳׳N, 87°12׳09׳׳E	6.0	
		129	23°33׳37׳׳N, 87°12׳27׳׳E	6.0	
		130	23°34׳45׳׳N, 87°09׳43׳׳E	5.5	
27	Pandabeswar Block	131	23°42׳37׳׳N, 87°13׳56׳׳E	6.5	6.0
		132	23°40׳28׳׳N, 87°16׳47׳׳E	6.5	
		133	23°38׳31׳׳N, 87°13׳01׳׳E	6.0	
		134	23°40׳24׳׳N, 87°16׳26׳׳E	6.0	
		135	23°40׳03׳׳N, 87°12׳44׳׳E	6.0	
28	Jamuria Block	136	23°46׳30׳׳N, 87°04׳14׳׳E	5.0	5.5
		137	23°41׳25׳׳N, 87°09׳07׳׳E	5.0	
		138	23°43׳19׳׳N, 87°07׳54׳׳E	5.5	
		139	23°38׳29׳׳N, 87°09׳28׳׳E	6.0	
		140	23°41׳50׳׳N, 87°11׳15׳׳E	5.5	
29	Raniganj Block	141	23°37׳27׳׳N, 87°02׳21׳׳E	6.0	5.5
		142	23°34׳26׳׳N, 87°07׳54׳׳E	6.0	
		143	23°37׳14׳׳N, 87°03׳47׳׳E	5.0	
		144	23°35׳06׳׳N, 87°06׳32׳׳E	5.5	
		145	23°36׳31׳׳N, 87°04׳49׳׳E	5.5	
30	Barabani Block	146	23°48׳20׳׳N, 86°58׳15׳׳E	4.5	5.5
		147	23°43׳45׳׳N, 87°00׳54׳׳E	5.5	
		148	23°47׳19׳׳N, 87°01׳37׳׳E	5.5	
		149	23°43׳52׳׳N, 86°58׳18׳׳E	6.0	
		150	23°46׳29׳׳N, 86°59׳25׳׳E	5.5	
31	Salanpur Block	151	23°50׳05׳׳N, 86°53׳36׳׳E	4.5	5.0
		152	23°45׳24׳׳N, 86°54׳51׳׳E	4.5	
		153	23°48׳31׳׳N, 86°54׳53׳׳E	5.5	
		154	23°43׳59׳׳N, 86°53׳44׳׳E	5.0	
		155	23°46׳53׳׳N, 86°51׳37׳׳E	5.0	
32	Durgapur Municipality Corporation	156	23°33׳26׳׳N, 87°20׳11׳׳E	6.0	6.0
		157	23°31׳31׳׳N, 87°15׳36׳׳E	6.0	
		158	23°32׳08׳׳N, 87°20׳46׳׳E	5.5	
		159	23°29׳02׳׳N, 87°18׳52׳׳E	5.5	
		160	23°29׳01׳׳N, 87°19׳35׳׳E	6.0	
33	Asansol Municipality Corporation	161	23°42׳06׳׳N, 87°03׳58׳׳E	5.0	5.5
		162	23°41׳58׳׳N, 86°53׳32׳׳E	5.0	
		163	23°39׳14׳׳N, 87°00׳14׳׳E	5.5	
		164	23°42׳58׳׳N, 86°49׳49׳׳E	5.5	
		165	23°38׳51׳׳N, 86°55׳58׳׳E	6.0	
34	Kalna Muicipality	166	23°10׳32׳׳N, 88°21׳01׳׳E	6.5	6.0
		167	23°09׳32׳׳N, 88°21׳36׳׳E	6.5	
		168	23°09׳55׳׳N, 88°21׳41׳׳E	6.0	
		169	23°09׳46׳׳N, 88°21׳05׳׳E	6.0	
		170	23°09׳35׳׳N, 88°22׳01׳׳E	6.0	
35	Katwa Muicipality	171	23°45׳46׳׳N, 87°41׳32׳׳E	7.0	6.5
		172	23°45׳46׳׳N, 87°41׳32׳׳E	7.0	
		173	23°45׳46׳׳N, 87°41׳32׳׳E	6.0	
		174	23°45׳46׳׳N, 87°41׳32׳׳E	6.5	
		175	23°45׳46׳׳N, 87°41׳32׳׳E	6.0	
36	Dainhat Muicipality	176	23°45׳46׳׳N, 87°41׳32׳׳E	7.0	6.0
		177	23°45׳46׳׳N, 87°41׳32׳׳E	5.5	
		178	23°45׳46׳׳N, 87°41׳32׳׳E	6.0	
		179	23°45׳46׳׳N, 87°41׳32׳׳E	6.0	
		180	23°45׳46׳׳N, 87°41׳32׳׳E	6.5	
37	Barddhaman Muicipality	181	23°45׳46׳׳N, 87°41׳32׳׳E	5.5	6.5
		182	23°45׳46׳׳N, 87°41׳32׳׳E	6.5	
		183	23°45׳46׳׳N, 87°41׳32׳׳E	7.0	
		184	23°45׳46׳׳N, 87°41׳32׳׳E	6.5	
		185	23°45׳46׳׳N, 87°41׳32׳׳E	6.5	
38	Memari Muicipality	186	23°45׳46׳׳N, 87°41׳32׳׳E	6.0	6.0
		187	23°45׳46׳׳N, 87°41׳32׳׳E	6.0	
		188	23°45׳46׳׳N, 87°41׳32׳׳E	6.5	
		189	23°45׳46׳׳N, 87°41׳32׳׳E	6.5	
		190	23°45׳46׳׳N, 87°41׳32׳׳E	6.0	
39	Guskara Muicipality	191	23°45׳46׳׳N, 87°41׳32׳׳E	7.0	6.5
		192	23°45׳46׳׳N, 87°41׳32׳׳E	7.0	
		193	23°45׳46׳׳N, 87°41׳32׳׳E	6.0	
		194	23°45׳46׳׳N, 87°41׳32׳׳E	6.5	
		195	23°45׳46׳׳N, 87°41׳32׳׳E	6.5	

**Table 2:** P^H^ range and nature of soil.

Soil P^H^	Nature of Soil
<4.5	Extremely acidic
4.5–5.0	Very strongly acid
5.1–5.5	Strongly acid
5.6–6.0	Moderately acid
6.1–6.5	Slightly acid
6.6–7.3	Neutral
7.4–7.8	Slightly alkaline
7.9–8.4	Moderately alkaline
8.5–9.0	Strongly alkaline
>9.1	Very strongly alkaline
