# Supplementary material for: Data on soil PH of Barddhaman district, India
Source: Data Brief. 2017 Apr 8;12:242–50. doi: 10.1016/j.dib.2017.03.046 (PMC5397573; doi:10.1016/j.dib.2017.03.046)
Supplement: Supplementary file 1 — Supplementary material [file mmc1.docx]

**Conflict of interest**: None.
